# Is Dorsal Vertical Double Plating an Effective Alternative to Volar Plating for Distal Radius Fractures With Dorsal Collapse?

**DOI:** 10.1111/os.70218

**Published:** 2025-12-03

**Authors:** Xu Tian, Bo Zhang, Junyang Liu, Lei Han, Peng Cui, Chao Pan, Genqiang Zheng, Bingshan Yan, Guangyu Wang, Lintao Liu, Jingming Dong, Qiang Yang

**Affiliations:** ^1^ Department of Orthopedics, Clinical College of Orthopedics Tianjin Medical University Tianjin China

**Keywords:** fracture fixation, plate, radius fractures

## Abstract

**Objective:**

Dorsal articular collapse in distal radius fractures presents unique fixation challenges. While volar locking plating (VLP) dominates current practice, dorsal vertical double plating (DVDP) offers direct biomechanical support but carries perceived tendon risks. This study compares DVDP versus VLP for dorsally collapsed comminuted fractures.

**Methods:**

A retrospective cohort of 106 patients (2022–2024) with AO type C2/C3 fractures received either VLP (*n* = 50) or DVDP (*n* = 56). General information encompassed gender, age, injured side, injury mechanism, AO classification, time from injury to surgery, operative time and complication profiles. Primary outcomes included 12‐month radiographic parameters (volar tilt, ulnar inclination, and radial height), wrist range of motion (ROM), functional scores (DASH, Gartland‐Werley), and complications. Continuous variables were compared using the Mann–Whitney *U* test. Categorical variables were analyzed with Pearson's *χ* ^2^ test.

**Results:**

The study cohort comprised 106 patients with dorsally collapsed distal radius fractures (VLP = 50, DVDP = 56). Baseline characteristics, including age (VLP median 59 years [IQR: 55–61.25] vs. DVDP 57 [53–61]), gender distribution (36% vs. 35.7% male), injury mechanism (72% vs. 71.4% falls), and AO classification (C3: 76% vs. 76.8%), showed no significant differences (all *p* > 0.05). At 12‐month follow‐up, all fractures achieved union with comparable radiographic outcomes: volar tilt (10° [8°–12°] vs. 10° [9°–12°]), ulnar inclination (22° [20°–23°] vs. 23° [22°–23°]), and radial height (11 mm [9–12] vs. 11 mm [10–12]) (all *p* > 0.05). Functional assessments revealed equivalent ranges of motion: dorsiflexion (69.5° [62°–76°] vs. 70° [68°–75°]), palmar flexion (68° [60°–70°] vs. 69.5° [66°–70°]), and rotation (pronation‐supination: 80° [67.75°–65°]/71.5° [61.5°–81.25°] vs. 75.5° [70°–82°]/75° [68°–80°]). Patient‐reported outcomes were similar: Gartland–Werley scores (5 [3–8] vs. 5 [3–7.75]) and DASH scores (12.5 [10–15.42] vs. 12.5 [12.5–15]) (all *p* > 0.05). Complication rates were comparable (VLP: 10% transient median neuropathy vs. DVDP: 12.5% tendon adhesions, *p* = 0.69), with all cases resolving conservatively within 3 months. Crucially, the DVDP group demonstrated zero tendon ruptures using tendon‐sparing techniques.

**Conclusion:**

DVDP demonstrates non‐inferior functional and radiographic outcomes to VLP for dorsally collapsed fractures. With meticulous technique—including intercompartmental approaches and low‐profile implants—DVDP eliminates historical tendon risks and serves as a viable surgical alternative.

## Graphical abstract

Dorsal vertical double plating shows equivalent functional outcomes to volar plating in dorsally collapsed distal radius fractures. With compartment‐sparing dissection and low‐profile implants, this approach achieved zero tendon ruptures, establishing its viability as a biomechanically effective alternative.

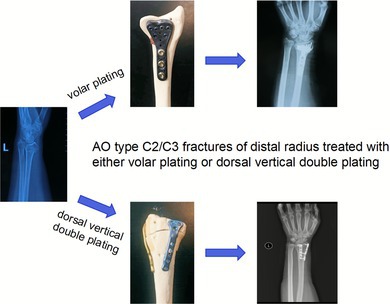

## 1 Introduction

Internal fixation has progressively become the mainstay for managing displaced distal radius fractures, superseding non‐operative methods. The choice of surgical approach is principally guided by fracture morphology, particularly the direction of displacement and the severity of comminution [1].

Although dorsal plating carries higher complication rates due to implant design constraints and dorsal anatomical considerations, it yields satisfactory functional outcomes [2, 3, 4]. With advancements in implant technology, volar locking plating (VLP) has gained prominence as the preferred approach. This technique mitigates dorsal approach‐related complications while achieving comparable therapeutic efficacy [1]. However, VLP is not without its own set of complications. Notably, issues such as flexor tendon irritation, potential median nerve symptoms and, if the plate is positioned improperly relative to the watershed line, tendon rupture remain concerns. Furthermore, specific risk factors, including advanced age (> 60 years), C‐type fractures, open fractures, and prolonged operative time (≥ 90 min), have been identified as independent predictors for postoperative soft tissue complications following VLP fixation [1, 5].

In contrast, dorsal plating, particularly for fractures with severe dorsal comminution or articular involvement, provides direct fragment control and robust dorsal support [2, 3, 4, 5]. While earlier dorsal implant designs were linked to higher rates of extensor tendon complications (e.g., tenosynovitis and attritional rupture), the evolution towards dorsal vertical double plating (DVDP) aims to address these shortcomings. This technique utilizes two low‐profile, anatomically contoured plates positioned orthogonally. The rationale is to enhance biomechanical stability while distributing the implant profile, thereby minimizing points of concentrated friction and irritation beneath the extensor tendon compartments. While this construct is theoretically advantageous, the specific complication profile and functional outcomes of DVDP, especially in direct comparison to the ubiquitous VLP, require clearer elucidation.

Current literature documents various plating strategies—volar, dorsal, and combined approaches—for distal radius fractures [5, 6, 7, 8, 9]. A substantial body of evidence, including systematic reviews and large cohort studies, supports the efficacy of VLP [4, 5]. Similarly, studies have reported on the outcomes of single dorsal plating and the application of dual plating in other anatomical regions, underscoring the broader relevance of dual‐plating concepts in orthopedics [7, 8, 9]. However, robust head‐to‐head comparative evidence focusing specifically on isolated volar locking plates versus the more specialized dorsal vertical double‐plating technique for specific, complex fracture patterns in the distal radius remains scarce. This gap is significant, as the choice between a volar approach with its specific risk profile and a modern dorsal double‐plating technique with its potential for reduced tendon complications lacks a strong, direct evidence base. Therefore, this study is necessary to provide a direct comparative analysis of the functional outcomes, radiographic results, and complication spectra—with particular attention to tendon‐related issues—between VLP and DVDP in the management of complex distal radius fractures.

The objectives of this study are to: (i) analyze the applications and technical considerations of VLP; (ii) make critical considerations for dorsal plating; (iii) expound key technical considerations for DVDP.

## 2 Materials and Methods

### 2.1 Subjects

A retrospective analysis included 106 patients with dorsally collapsed comminuted distal radius fractures (AO type C2/C3 fractures) treated surgically at our institution between January 2022 and December 2024. The study was approved by the ethics committee of Tianjin hospital (2025 Medical Ethics Review 091). Consent was obtained from all patients, and the study conformed to the provisions of the Declaration of Helsinki (as revised in Brazil in 2013). The cohort comprised two groups: VLP group and DVDP group. All operations were performed by the same experienced surgeon team (X.T., M.D.; B.Z., M.D.; and J.Y.L., M.D.).

*Inclusion criteria*: (i) patients over 18 years old; (ii) acute wrist injury (< 1 week); (iii) AO type C2/C3 fractures of distal radius; (iv)treatment with open reduction and internal fixation of the distal radius; (v) internal fixation is either volar locking plate or DVDP; (vi) follow‐up at least 12 months.

*Exclusion criteria*: (i) previous injury on the affected wrist; (ii) pathological fractures; (iii) fracture of the distal ulnar that should be fixed; (iv) pre‐existing wrist dysfunction.

### 2.2 | General Information

General information encompassed gender, age, injured side, injury mechanism, AO classification, time from injury to surgery, operative time and complication profiles. At the 12‐month follow‐up, comprehensive assessments included radiographic evaluation through standard posteroanterior and lateral wrist radiographs quantifying volar tilt, ulnar inclination, and radial height; functional range of motion (ROM) measurements assessing active dorsiflexion, palmar flexion, ulnar deviation, radial deviation, pronation, and supination; along with validated functional scoring using the Gartland–Werley system and DASH questionnaire.

### 2.3 Surgical Procedure

**FIGURE 1:**
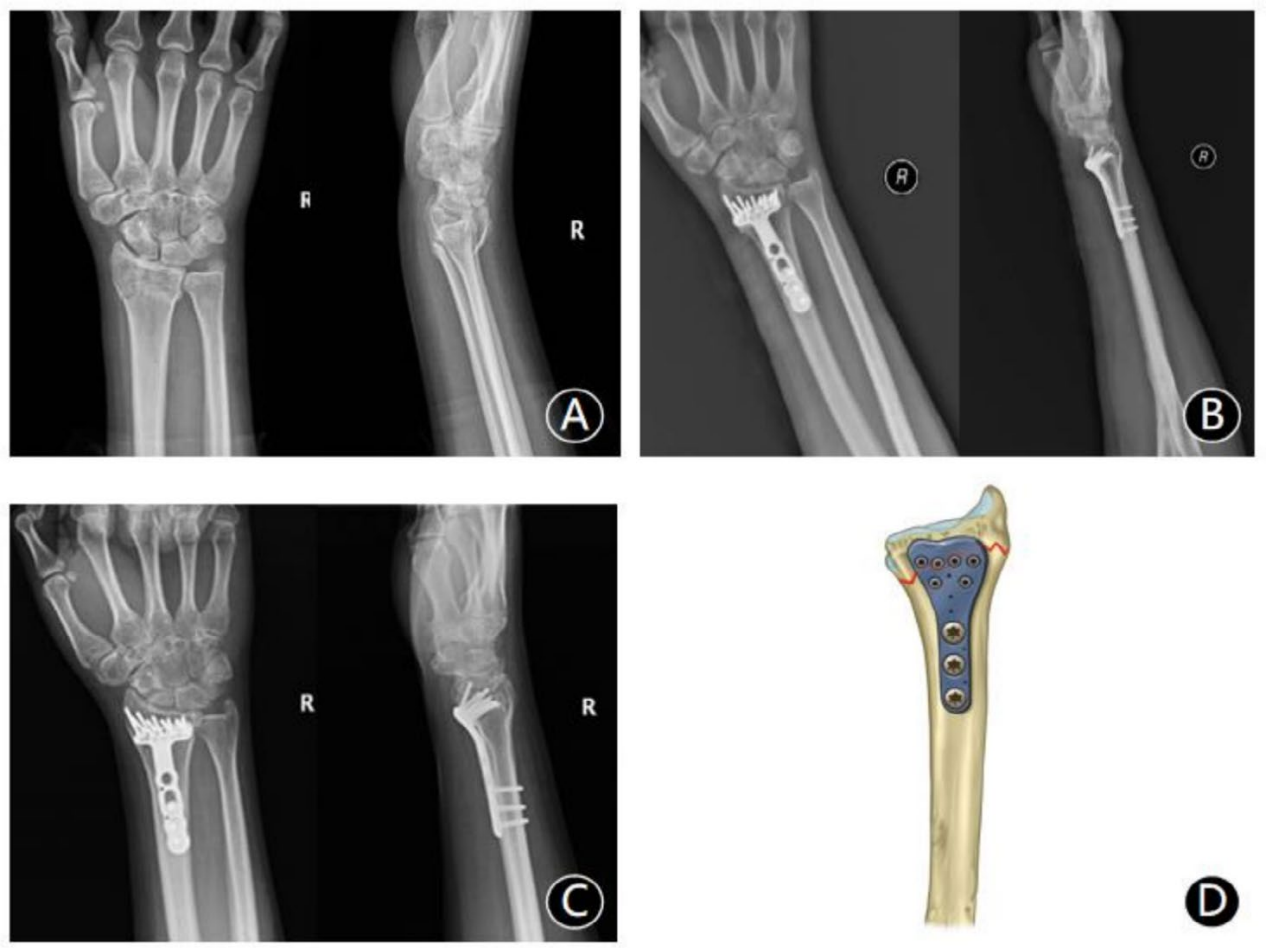
A 58‐year‐old male with dorsally collapsed distal radius fracture. (A) Preoperative radiographs: Posteroanterior (PA) and lateral views demonstrate severe dorsal articular collapse (> 5 mm displacement) with radial shortening. (B) Postoperative radiographs: Following volar locking plate fixation, anatomical restoration of volar tilt and radial height is achieved. (C) Fracture union with preserved anatomical reduction observed on 12‐month postoperative radiographs. (D) Surgical schematic: Optimal plate positioning along the watershed line with distal screws engaging the subchondral bone.

*Volar plating group*: A longitudinal volar incision was made over the distal radius. The approach was developed between the flexor carpi radialis and the radial artery. The pronator quadratus was elevated to expose the fracture site. After debridement and reduction, temporary K‐wire fixation was applied. Reduction adequacy was verified under C‐arm fluoroscopy. An anatomically contoured VLP (2.7 mm volar locking plate for distal radius, BEST, China) was then applied to the distal radius, with screws inserted in proximal and distal fragments. Final radiographs confirmed satisfactory reduction and hardware placement. The wound was irrigated, the pronator quadratus repaired, a drain placed, and the incision closed (Figure 1).

*Key technical considerations*: To ensure safety and efficacy, several technical points were emphasized. The interval between the flexor carpi radialis and the radial artery was reliably identified by retracting the former ulnarly and the latter radially. During the approach, a small cuff of the pronator quadratus was left on the radius to facilitate its eventual repair. Critical to preventing flexor tendon complications was the placement of the plate proximal to the watershed line. Finally, a robust, anatomic repair of the pronator quadratus was performed to create a biological barrier between the implant and the flexor tendons.

**FIGURE 2:**
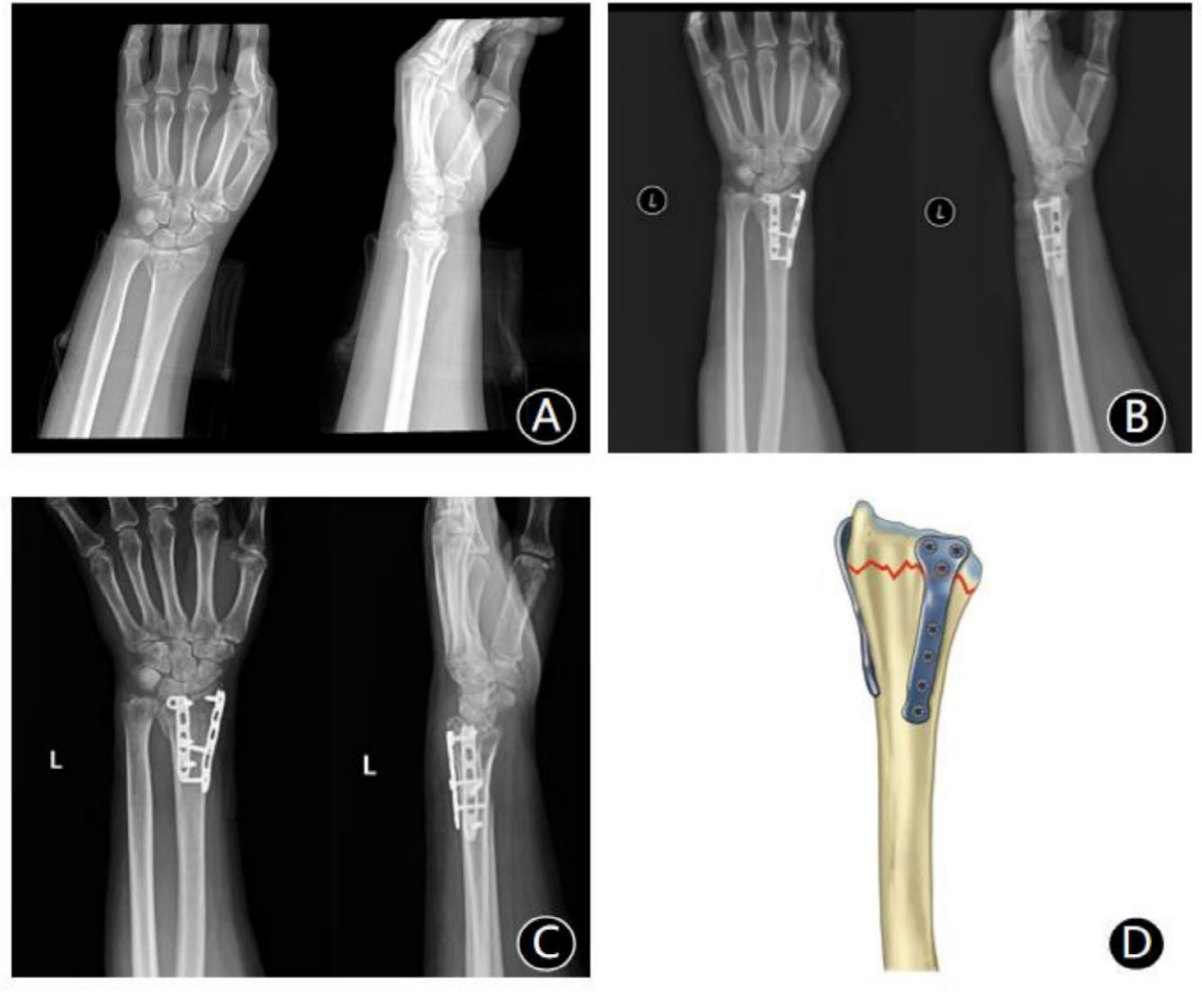
A 58‐year‐old female with dorsally collapsed distal radius fracture. (A) Preoperative radiographs: Posteroanterior (PA) and lateral views reveal severe dorsal articular collapse (> 4 mm displacement) with radial shortening and comminution. (B) Postoperative radiographs: Following DVDP, anatomical restoration is achieved. (C) Fracture union with preserved anatomical reduction observed on 12‐month postoperative radiographs. (D) Surgical schematic: Plate positioning on radial (between 1st and 2nd compartments) and intermediate (between 3rd and 4th compartments) columns with subperiosteal dissection.

*DVDP group*: A dorsal incision was made ulnar to Lister's tubercle. The approach utilized intercompartmental intervals while minimizing tendon sheath violation (particularly for the extensor pollicis longus). The radial column was exposed between the 1st (abductor pollicis longus and extensor pollicis brevis) and 2nd (extensor carpi radialis longus/brevis) dorsal compartments. The intermediate column was accessed between the 3rd (extensor pollicis longus) and 4th (extensor digitorum communis and extensor indicis proprius) compartments. Following fracture debridement and reduction, temporary K‐wire fixation was applied. Reduction was confirmed by C‐arm fluoroscopy. Dorsal vertical plates (2.7 mm mini‐locking plate, BEST, China) were applied to the radial and intermediate columns, with screws securing proximal and distal fragments. After radiographic confirmation, the wound was irrigated, a drain was inserted, and the incision was closed (Figure 2).

*Key technical considerations*: Specific techniques were employed to mitigate the historically higher risk of extensor tendon complications. The extensor pollicis longus tendon was identified and retracted radially to safely access the intermediate column. The two plates were positioned to form a low‐profile, orthogonal (90–90) construct without overlapping, thereby minimizing overall implant bulk. Meticulous attention was paid to screw length to secure subchondral purchase without violating the volar cortex. A critical final step involved repairing the extensor retinaculum, and where possible, suturing a portion of it deep to the plates to interpose a soft tissue layer between the implants and the tendons.

### 2.4 Postoperative Management

All patients received splint immobilization postoperatively. They started physical therapy on the day after surgery. Active finger flexion/extension and limited passive wrist motion (20° flexion to 20° dorsiflexion) commenced on postoperative day 1, with rotational movements restricted. Wound inspection and drain removal were performed on day 2. Sutures were removed at 2 weeks. Passive ROM was progressively increased to 35° flexion/dorsiflexion by week 2. Passive wrist rotation exercises with graded range increment were initiated at 4 weeks. Active mobilization was progressively introduced according to radiographic evidence of fracture union. The therapy continued until the patients reached their maximum improvements.

### 2.5 Outcome Measures

General information encompassed gender, age, injured side, injury mechanism, AO classification, time from injury to surgery, operative time and complication profiles. At the 12‐month follow‐up, comprehensive assessments included radiographic evaluation through standard posteroanterior and lateral wrist radiographs quantifying volar tilt (°), ulnar inclination (°), and radial height (mm); functional ROM measurements assessing active dorsiflexion, palmar flexion, ulnar deviation, radial deviation, pronation, and supination (°); alongside validated functional scoring using the Gartland–Werley system [10] and Disability of Arm, Shoulder and Hand (DASH) questionnaire [11].

### 2.6 Statistical Analysis

Statistical analyses were performed using SPSS 23.0 (IBM Corp., Armonk, NY, USA). Continuous variables—including wrist ROM (dorsiflexion, palmar flexion, ulnar deviation, radial deviation, pronation, and supination), functional scores (Gartland–Werley, DASH), radiographic parameters (volar tilt, ulnar inclination, radial height), age, time from injury to surgery and operative time‐demonstrated non‐normal distribution in both groups (Shapiro–Wilk scores were 0.953, 0.915, 0.910, 0.960, 0.947, 0.952, 0.939, 0.914, 0.963, 0.891, 0.948, 0.957, 0.859, and 0.931, respectively, all *p* < 0.05). These were consequently expressed as median with interquartile range (IQR; P25, P75) and compared using the Mann–Whitney *U* test. Categorical variables (gender, injured side, injury mechanism, AO classification, and complication) were analyzed with Pearson's *χ* ^2^ test. Statistical significance was defined as *p* < 0.05.

## 3 Results

### 3.1 General Information

The study cohort comprised 106 patients with dorsally collapsed distal radius fractures. In the VLP group (*n* = 50), patients had a median age of 59 years (IQR: 55–61.25) with 18 males (36%) and 32 females (64%), including 29 right‐side (58%) and 21 left‐side injuries (42%). The DVDP group (*n* = 56) exhibited a median age of 57 years (IQR: 53–61) with 20 males (35.7%) and 36 females (64.3%), featuring 32 right‐side (57.1%) and 24 left‐side injuries (42.9%). All fractures were closed, primarily caused by falls (VLP: *n* = 36, 72%; DVDP: *n* = 40, 71.4%) and traffic accidents (VLP: *n* = 14, 28%; DVDP: *n* = 16, 28.6%). According to AO classification, type C3 fractures predominated (VLP: *n* = 38, 76%; DVDP: *n* = 43, 76.8%) over type C2 (VLP: *n* = 12, 24%; DVDP: *n* = 13, 23.2%). Median time from injury to surgery was 4 days (IQR: 3–4.25) for VLP and 3 days (IQR: 3–5) for DVDP. Operative time was 42.5 min (IQR: 40.0–46.0) for VLP and 43.0 min (IQR: 40.0–47.5) for DVDP. No statistically significant differences were observed in baseline characteristics between groups (all comparisons *p* > 0.05; Table 1).

**TABLE 1:** General information.

Characteristic	VLP group	DVDP group	*U* value/*χ* ^2^	*p*
Gender (M/F)	18/32	20/36	0.001	0.976
Age (years°)	59 (55, 61.25)	57 (53, 61)	1176.5	0.156
Injured side (R/L)	29/21	32/24	0.008	0.929
Injury mechanism (fall/traffic accident)	36/14	40/16	0.004	0.948
AO classification (C2/C3)	12/38	13/43	0.1	0.752
Time from injury to surgery (days)	4 (3, 4.25)	3 (3, 5)	1318	0.585
Operative time (min)	42.5 (40.0, 46.0)	43.0 (40.0, 47.5)	1367.5	0.836

### 3.2 Radiographic Outcome

By the 12‐month follow‐up, all fractures had achieved radiographic union with comparable measurements between groups: the VLP cohort demonstrated a median volar tilt of 10° (IQR: 8–12), ulnar inclination of 22° (IQR: 20–23), and radial height of 11 mm (IQR: 9–12), while the DVDP group showed corresponding values of 10° (IQR: 9–12), 23° (IQR: 22–23), and 11 mm (IQR: 10–12), respectively. No statistically significant intergroup differences were observed in these radiographic parameters (all *p* > 0.05; Table 2).

**TABLE 2:** Radiographic measurement.

Characteristic	VLP group	DVDP group	*U* value	*p*
Volar tilt (°)	10 (8, 12)	10 (9, 12)	1179	0.156
Ulnar inclination (°)	22 (20, 23)	23 (22, 23)	1271.5	0.395
Radial height (mm)	11 (9, 12)	11 (10, 12)	1280	0.439

### 3.3 Functional Outcome

All surgical wounds healed uneventfully. At the 12‐month assessment, wrist ROM demonstrated comparable results between groups: the VLP cohort showed median dorsiflexion of 69.5° (IQR: 62–76), palmar flexion of 68° (IQR: 60–70), ulnar deviation of 24° (IQR: 20–25.25), radial deviation of 18.5° (IQR: 16–20.25), pronation of 80° (IQR: 67.75–65), and supination of 71.5° (IQR: 61.5–81.25), while the DVDP group exhibited corresponding values of 70° (IQR: 68–75), 69.5° (IQR: 66–70), 25° (IQR: 22.25–26), 20° (IQR: 18–21), 75.5° (IQR: 70–82), and 75° (IQR: 68–80), respectively. No statistically significant differences were observed in any motion parameters (all *p* > 0.05; Table 3).

**TABLE 3:** Wrist range of motion.

Characteristic	VLP group	DVDP group	*U* value	*p*
Dorsiflexion (°)	69.5 (62, 76)	70 (68, 75)	1263.5	0.386
Palmar flexion (°)	68 (60, 70)	69.5 (66, 70)	1155.5	0.117
Ulnar deviation (°)	24 (20, 25.25)	25 (22.25, 26)	1124	0.077
Radial deviation (°)	18.5 (16, 20.25)	20 (18, 21)	1127	0.08
Pronation (°)	80 (67.75, 65)	75.5 (70, 82)	1294	0.501
Supination (°)	71.5 (61.5, 81.25)	75 (68, 80)	1326	0.639

### 3.4 Functional Score and Complication

At the 12‐month follow‐up, functional assessments revealed comparable outcomes: the VLP group demonstrated a median Gartland‐Werley score of 5 (IQR: 3–8) and DASH score of 12.5 (IQR: 10–15.42), while the DVDP group showed corresponding scores of 5 (IQR: 3–7.75) and 12.5 (IQR: 12.5–15). Complication rates were similarly distributed with five cases (10%) in the VLP group versus seven cases (12.5%) in the DVDP group. No statistically significant differences were observed in these outcome measures (all *p* > 0.05; Table 4).

**TABLE 4:** Functional score and complication.

Characteristic	VLP group	DVDP group	*U* value/*χ* ^2^	*p*
Gartland–Werley score	5 (3, 8)	5 (3, 7.75)	1353	0.765
DASH questionnaire	12.5 (10, 15.42)	12.5 (12.5, 15)	1368.5	0.84
Complication	5	7	0.164	0.685

In the VLP group, five patients (10%) developed transient median neuropathy symptoms, likely attributable to intraoperative traction. The DVDP group had seven cases (12.5%) of tendon adhesions. All complications resolved completely within 3 months postoperatively through neurotrophic medication and structured rehabilitation protocols.

## 4 Discussion

Our study cohort comprised 106 patients with dorsally collapsed distal radius fractures, and treated with either VLP (*n* = 50) or DVDP (*n* = 56). At 12‐month follow‐up, all fractures achieved union with comparable radiographic outcomes and functional assessments revealed equivalent ranges of motion; patient‐reported outcomes were similar. Our primary findings indicate that DVDP is an effective alternative to volar plating for distal radius fractures with dorsal collapse.

### 4.1 Volar Plating: Applications and Technical Considerations

Distal radius fractures represent common upper extremity injuries. Those involving comminution of the dorsal intermediate column often feature an intact volar cortex with dorsal fragmentation. Insufficient dorsal support may lead to reduction failure, necessitating surgical intervention with specific fixation requirements: robust stabilization of dorsal intermediate column fragments must be achieved.

Advances in volar locking plate design—particularly angular‐stable implants—have expanded indications to include dorsally comminuted fractures [12]. The volar approach requires meticulous protection of the radial artery and median nerve. Plate placement must adhere strictly to implant‐specific principles, with most contemporary systems following the watershed line concept. When anatomical reduction and proper plate positioning are achieved, intra‐articular screw penetration can be reliably avoided [13].

Our volar plating cohort experienced no plate‐related complications, though transient median neuropathy occurred. We believe that the occurrence of transient median nerve symptoms is due to over‐retraction during surgery. For relatively straightforward distal radius fractures, the surgical incision is often smaller, resulting in excessive traction on the tissues, which leads to median nerve symptoms. Such complications may be preventable with refined technique. Perregaard et al. [14] demonstrated that the distal screws in volar plating can be directed superoproximally to engage the physiological 12° volar tilt, thereby providing subchondral support to prevent fragment collapse and secondary displacement. This anatomical advantage also minimizes radiocarpal joint penetration.

Laane et al. [15] further established that appropriate volar plate placement eliminates tendon‐related complications while maintaining reduction integrity without hardware failure. However, for dorsally comminuted fractures, achieving sufficient dorsal support through volar plating requires precise bicortical engagement of the ulnar‐most screws without dorsal cortex protrusion—a technically demanding standard particularly challenging in osteoporotic bone. In such scenarios, direct dorsal intermediate column plating offers a mechanically advantageous and technically efficient alternative.

### 4.2 Critical Considerations for Dorsal Plating

Dorsal plating is indicated for distal radius fractures with severe dorsal involvement or dorsal instability, providing direct and effective fixation. However, tendon rupture or adhesion remains the most recognized complication of dorsal approaches. Multiple studies report elevated tendon‐related complication rates following dorsal plating [16, 17], including documented cases of attritional tendon rupture due to hardware prominence—a concern potentially related to implant material and design.

The application of a dorsal plate mandates a sophisticated approach to mitigate its inherent risk of extensor tendon pathology. The fundamental principle revolves around transforming the implant from a potential irritant into a seamless internal stabilizer. This is achieved through a trifecta of technical precision: selection of an appropriately low‐profile implant, its meticulous placement entirely beneath the extensor tendon compartments, and the exacting control of screw length to secure subchondral bone in the volar cortex without any dorsal prominence. Violation of any of these principles significantly elevates the probability of postoperative tenosynovitis, tendon adhesion, or ultimately, attritional rupture.

### 4.3 Surgical Tips for DVDP

The DVDP technique is distinct from the application of a single, large dorsal plate. It is characterized by the use of two low‐profile locking plates positioned in an orthogonal construct. This strategic arrangement achieves the required biomechanical stability for complex fractures, while its low‐profile nature significantly reduces the risk of soft tissue irritation and tendon impingement on the dorsal aspect. In our cohort, no tendon ruptures occurred in patients treated with DVDP. We attribute this to meticulous surgical technique and early postoperative rehabilitation. Labrum et al. [18] identified Lister's tubercle as a critical site for tendon irritation with dorsal plating, recommending prophylactic extensor pollicis longus (EPL) release to prevent rupture. Key technical considerations include: Utilizing low‐profile plates with countersunk screws to minimize tendon contact; Accessing the radial column via the 1st‐2nd extensor compartment interval; Approaching the intermediate column through the 3rd‐4th compartment interval; Avoiding unnecessary tendon dissection (particularly of the EPL); Early functional mobilization remains essential for preventing tendon adhesions.

### 4.4 Limitations and Strengths

This study's retrospective single‐center design introduces inherent selection biases despite statistical adjustments, with non‐randomized technique allocation potentially confounding fracture complexity patterns. Outcomes reflect a specialized surgical team's expertise, limiting generalizability to surgeons navigating the learning curve of compartment‐sparing dissection. Although adequately powered for primary functional endpoints, the cohort size was insufficient to detect rare complications. The 12‐month follow‐up captures functional recovery but falls short of evaluating late implant sequelae—particularly critical for dorsal approaches where attritional tendon pathology may manifest beyond 24 months. Additionally, unquantified adherence variations in home‐based rehabilitation may influence ROM outcomes. Crucially, these limitations do not invalidate our demonstration of DVDP's non‐inferiority to VLP. Future multicenter RCTs with protocolized training, extended surveillance, and quantified rehabilitation metrics are warranted.

This study, while having certain limitations, demonstrates notable strengths. First, it provides the first detailed comparative analysis between primary DVDP and volar plating—a comparison rarely documented in existing literature. Furthermore, by comprehensively evaluating clinical outcomes including ROM and functional scores, this research facilitates a systematic understanding of patients' overall postoperative recovery.

## 5 Conclusion

Our findings demonstrate comparable functional outcomes (DASH, Gartland–Werley scores) between VLPs and DVDP for dorsally displaced comminuted distal radius fractures. This clinical equivalence is logically consistent with the absence of significant complication rate differences. When surgeons possess proficiency in dorsal approaches, DVDP represents a viable alternative to conventional volar plating for managing these complex fractures.

## Author Contributions

**Xu Tian:** conceptualization, methodology, data curation, writing – original draft, writing – review and editing, software, resources, validation, visualization. **Bo Zhang:** conceptualization, methodology, writing – original draft, resources, visualization. **Junyang Liu:** data curation, investigation. **Lei Han** and **Peng Cui:** investigation, data curation. **Chao Pan, Genqiang Zheng,** and **Bingshan Yan:** formal analysis. **Guangyu Wang, Lintao Liu,** and **Jingming Dong:** data curation. **Qiang Yang:** methodology, project administration, supervision. All authors read and approved the final article.

## Funding

The authors have nothing to report.

## Conflicts of Interest

The authors declare no conflicts of interest.

## Data Availability

The data that support the findings of this study are available on request from the corresponding author. The data are not publicly available due to privacy or ethical restrictions.
